# High Tuberculosis Prevalence in a South African Prison: The Need for Routine Tuberculosis Screening

**DOI:** 10.1371/journal.pone.0087262

**Published:** 2014-01-30

**Authors:** Lilanganee Telisinghe, Katherine L. Fielding, Justin L. Malden, Yasmeen Hanifa, Gavin J. Churchyard, Alison D. Grant, Salome Charalambous

**Affiliations:** 1 Aurum Institute, Johannesburg, South Africa; 2 CAPRISA, University of KwaZulu-Natal, Durban, South Africa; 3 London School of Hygiene & Tropical Medicine, London, United Kingdom; 4 School of Public Health, University of Witwatersrand, Johannesburg, South Africa; Institute of Infectious Diseases and Molecular Medicine, South Africa

## Abstract

**Background:**

Tuberculosis is a major health concern in prisons, particularly where HIV prevalence is high. Our objective was to determine the undiagnosed pulmonary tuberculosis (“undiagnosed tuberculosis”) prevalence in a representative sample of prisoners in a South African prison. In addition we investigated risk factors for undiagnosed tuberculosis, to explore if screening strategies could be targeted to high risk groups, and, the performance of screening tools for tuberculosis.

**Methods and Findings:**

In this cross-sectional survey, male prisoners were screened for tuberculosis using symptoms, chest radiograph (CXR) and two spot sputum specimens for microscopy and culture. Anonymised HIV antibody testing was performed on urine specimens. The sensitivity, specificity and predictive values of symptoms and investigations were calculated, using *Mycobacterium tuberculosis* isolated on sputum culture as the gold standard.

From September 2009 to October 2010, 1046 male prisoners were offered enrolment to the study. A total of 981 (93.8%) consented (median age was 32 years; interquartile range [IQR] 27–37 years) and were screened for tuberculosis. Among 968 not taking tuberculosis treatment and with sputum culture results, 34 (3.5%; 95% confidence interval [CI] 2.4–4.9%) were culture positive for *Mycobacterium tuberculosis*. HIV prevalence was 25.3% (242/957; 95% CI 22.6–28.2%). Positive HIV status (adjusted odds ratio [aOR] 2.0; 95% CI 1.0–4.2) and being an ex-smoker (aOR 2.6; 95% CI 1.2–5.9) were independently associated with undiagnosed tuberculosis. Compared to the gold standard of positive sputum culture, cough of any duration had a sensitivity of 35.3% and specificity of 79.6%. CXR was the most sensitive single screening modality (sensitivity 70.6%, specificity 92.2%). Adding CXR to cough of any duration gave a tool with sensitivity of 79.4% and specificity of 73.8%.

**Conclusions:**

Undiagnosed tuberculosis and HIV prevalence was high in this prison, justifying routine screening for tuberculosis at entry into the prison, and intensified case finding among existing prisoners.

## Introduction

Prisoners worldwide are at a disproportionately high risk of tuberculosis [1], [2] as well as HIV infection [3], a potent risk factor for tuberculosis. Prisoners may originate from deprived communities with high rates of tuberculosis. Within prisons, overcrowding, poor ventilation and nutrition, limited health services and a mobile population can contribute to ongoing disease transmission [4], [5]. Studies from Sub-Saharan African prisons suggest that 0.7% to 5.8% prisoners have undiagnosed active tuberculosis [4], [6]–[12], with multi-drug resistant (MDR) tuberculosis in 9.5% of isolates in one survey from Zambia [9].

In South Africa, the estimated adult prevalence of HIV infection was 18% in 2009 [13] and tuberculosis incidence was estimated at 981/100,000 in 2010 [14]; with an HIV prevalence of 60% among tuberculosis patients [14]. South Africa has the third highest incarceration rate in Africa with 316 prisoners per 100,000 population in 2011 [15]. However, there are no representative data regarding the prevalence of tuberculosis [16] or HIV among South Africa’s prisoners [17]–[19].

When a high prevalence of tuberculosis is anticipated, screening to identifying those requiring further investigation is important. A screening strategy should be easy to implement, and the tool/s used should have high sensitivity. This is usually followed with a diagnostic test of high specificity to identify patients with tuberculosis. International guidelines concerning tuberculosis control in prisons [5], [20], [21] recommend systematic screening of new entrants using a standardised symptom screen and if resources permit, especially in high tuberculosis prevalence settings, chest radiography to identify those requiring further investigation. Additional strategies advocated include sputum microscopy at entry [21] in high burden settings and periodic screening of all prisoners [5], [20]–[22]. The suggested screening symptoms differ between guidelines. A recent systematic review [2] indicated that only 3/52 countries included, all from low and middle income settings, used the World Health Organization (WHO) scoring system recommended for prisons [20], which has reported sensitivities ranging from 58% to 64% for bacteriologically confirmed tuberculosis [23], [24]. New WHO guidelines for intensified case finding (ICF) among people with HIV recommend screening with any one of current cough, fever, weight loss or night sweats [25], but data on the performance of this tool within prisons is lacking. Studies from prisons investigating screening tools to identify active tuberculosis are restricted, as not all comparator screening methods were used on all participants regardless of symptoms [23], [24], [26], [27]. Determining the sensitivity and specificity of individual tools, including symptoms in this setting, has therefore been limited. There is an urgent need to evaluate how best to screen prisoners for active tuberculosis.

We conducted a cross-sectional survey of prisoners in South Africa’s largest prison, in Johannesburg, housing up to 13,000 prisoners. At the time of the study, new entrants were screened for tuberculosis by enquiring on possible symptoms, which was not performed in a standardised manner; periodic screening and chest radiography were not routine. Prisoners with drug-resistant tuberculosis were segregated, but those who were sputum smear-positive for acid fast bacilli were not. HIV counselling and testing (HCT) and antiretroviral therapy (ART) have been available since 2007. The aim of the study was to determine the prevalence of active undiagnosed pulmonary tuberculosis (henceforth called undiagnosed tuberculosis) in the prison. In addition risk factors for undiagnosed tuberculosis were explored in order to investigate if screening strategies could be targeted to high risk groups; and the performance of symptom combinations and standard investigations in the diagnosis of tuberculosis was evaluated in this prison.

## Methods

### Ethics Statement

The study was approved by the Research Ethics Committees of the Department of Correctional Services, South Africa, the University of KwaZulu-Natal, South Africa and the London School of Hygiene & Tropical Medicine, UK; and the Centers for Disease Control (CDC) Institutional Review Board. The study was also approved by the Office for Human Research Protections (OHRP), USA.

All participants gave voluntary written informed consent, or verbal consent which was witnessed if unable to write. Study procedures were explained to the participants by trained research nurses, and participant information sheets were provided for participants to read. Participant information sheets were available in the most common local languages. Comprehension was ascertained prior to obtaining consent by asking participants to repeat back in their own words, their understanding of the study and procedures if they took part. If verbal consent was being provided, witnesses who were neither prison nor study staff were chosen by the participants; the participant’s thumbprint and the witness’s signature were used on the consent form. Participants with diminished capacity were not recruited to the study. Consent was specifically sought to undertake anonymised urine HIV testing from each study participant. All research ethics committees, review boards and the OHRP were aware and approved HIV testing procedures.

Consent and participation in the study was voluntary. Participants were able to refuse to take part, with no consequences to their healthcare or any other services as a result of this.

### Study Design and Population

The study site was the prison block housing sentenced male prisoners. Enrolment to the study took place between September 2009 and October 2010. Two groups of prisoners were approached to take part. The first was a simple random sample of prisoners who had been incarcerated for at least six months (“currently incarcerated”) at the start of the study. The second group were a consecutive sample of “newly-sentenced” prisoners who were entering the study site after having been sentenced in court. All those with an expected stay less than three months were excluded, as follow-up of medical records could not be assured.

### Study Procedures

All participants underwent a standardised symptom questionnaire, chest radiography (assessed by two readers using a standardised tool), and provided two spot sputum specimens for smear and mycobacterial culture. Urine for anonymised HIV testing was collected from those consenting; which was used only for study purposes. Results of anonymised urine HIV testing, was not given to study participants. For those wanting to know their HIV status, HCT, with linkage to appropriate care as necessary, was offered.

Tuberculosis suspects, defined as participants with clinical (respiratory symptoms, fever, night sweats, loss of appetite, lethargy, unintentional weight loss, temperature ≥38^o^C) and/or radiological features of tuberculosis, and/or positive sputum smear (any grade) or culture, were referred to the prison medical services for further management. All prisoners referred were managed according to the South African National Tuberculosis Control Programme Practical Guidelines [28]. They were reviewed one month later with repeat symptom screen, chest radiography, and two further sputum samples for smear and culture. Prison medical records of all prisoners enrolled to the study, without a diagnosis of tuberculosis during the study, were reviewed at three months post-enrolment to ascertain any tuberculosis diagnoses missed.

### Laboratory Methods

Sputum specimens underwent fluorochrome microscopy and liquid culture, at the National Health Laboratory Services of South Africa. Ten percent of all microscopy slides were double read, for quality control purposes. Positive cultures underwent speciation using GenoType® Mycobacterium CM (Hain Lifescience, Nehren, Germany) and drug susceptibility testing for isoniazid and rifampicin.

Anonymised urine samples were tested for HIV antibodies using the MAXIM HIV-1 urine EIA (Maxim Biomedical Inc, MD, USA). The urine EIA has a reported sensitivity of 99% and specificity of 90% among high HIV risk populations [29].

### Definitions

Tuberculosis was classified as *definite, probable and possible cases*.

1. *Definite cases* were sputum culture positive for *M. Tuberculosis* with compatible clinical or radiological features (as assessed by one or both readers), or additional microbiological confirmation (any grade of smear or further positive culture).
2. *Probable cases* were those with one culture positive for *M. tuberculosis* without compatible clinical or radiological features or smear positive grade 1+ or more, with or without compatible clinical or radiological features (as assessed by one or both readers).
3. *Possible* cases were culture-negative with either classical radiological features (pleural effusion, cavitation, or upper lobe changes, requiring two readers’ consensus) *or* one or more scanty positive smear.

Compatible radiological features were any feature suggestive of tuberculosis on chest radiography. Compatible clinical features consisted of at least one of cough >2weeks, night sweats, unintentional weight loss or temperature ≥38^o^C. All results (from enrolment and/or follow-up) could contribute to tuberculosis case definitions. Undiagnosed tuberculosis was defined as definite or probable tuberculosis in a person not taking treatment at enrolment.

An ex-smoker was defined as having smoked at least 100 cigarettes, but was not smoking at study enrolment. A current smoker was defined as having smoked at least 100 cigarettes and was smoking at study enrolment.

### Statistical Analysis

The target sample size was 1000 participants (new recruits and currently incarcerated combined), aiming to determine a tuberculosis prevalence of 2% with precision of 0.9% assuming a 95% confidence interval (CI). Data were analysed using Stata 11.0 (Stata Corporation, Texas, USA). Using logistic regression, unadjusted and adjusted odds ratios (OR) were calculated, with p-values from the likelihood ratio test. Continuous variables were categorised for analysis. Age was categorised into three levels (20–29 years, 30–34 years and >35years) and all others were dichotomised around the median. Factors associated with undiagnosed tuberculosis in univariable analysis with p<0.2 were considered for inclusion in a multivariable model. From the shortlist of variables considered for inclusion, only those most strongly associated with undiagnosed tuberculosis were included. When variables were strongly associated with each other (e.g. alcohol and smoking), only the variable more strongly associated with the outcome was included. As previous tuberculosis treatment was likely to be on the causal pathway between HIV infection and undiagnosed tuberculosis, it was not considered for the multivariable model. Sensitivities, specificities, negative and positive predictive values for combinations of screening methods were calculated. Only study screening tests performed at enrolment were used to evaluate screening methods.

## Results

**Figure 1:**
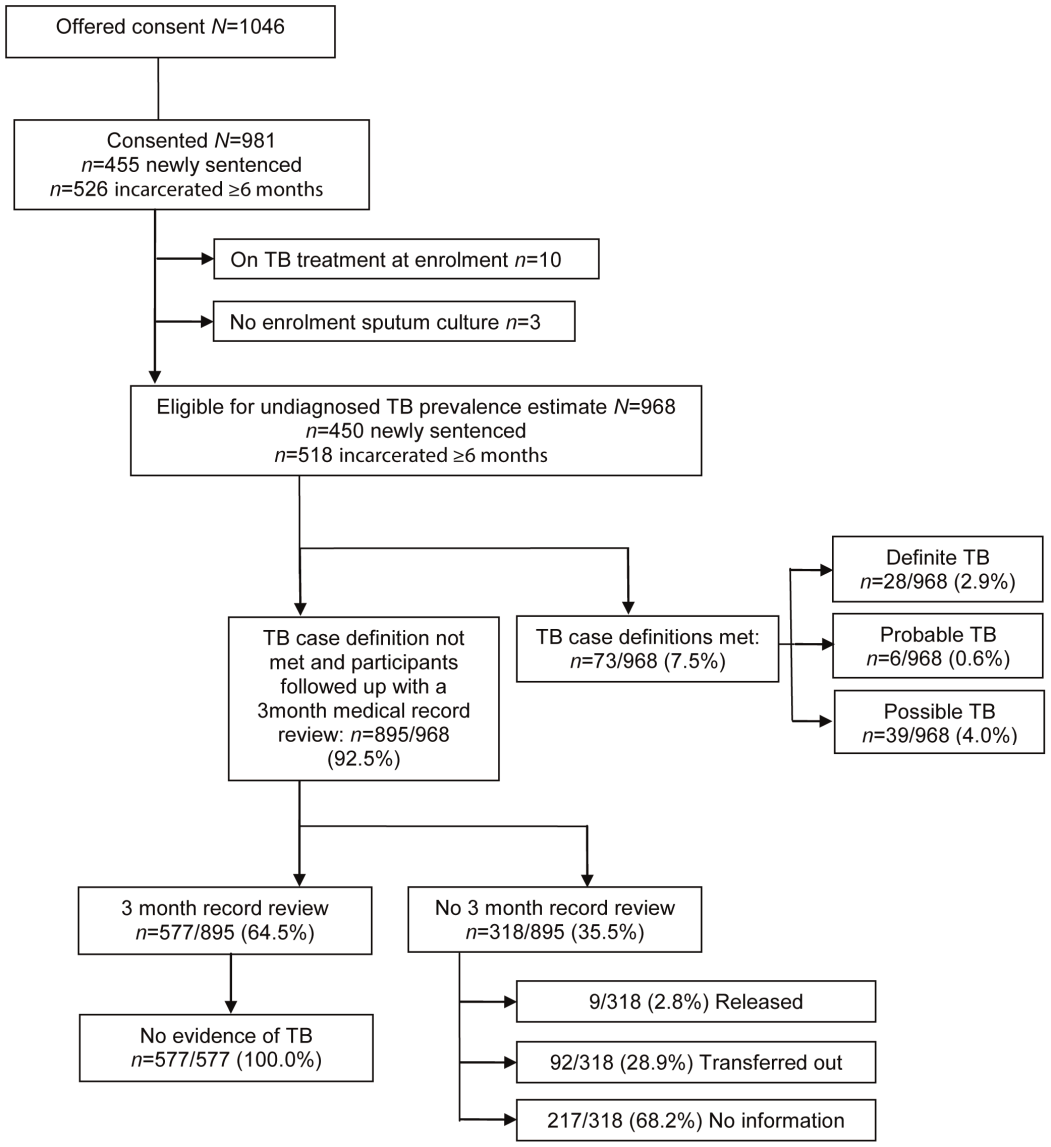
Study inclusions, losses to follow-up and tuberculosis outcomes. TB = Tuberculosis.

Figure 1 summarises study inclusions, losses to follow-up, and tuberculosis outcomes. A total of 1046 prisoners were offered consent to the study, with 981/1046 (93.8%) consenting. Of those consented 526 (53.6%) were currently incarcerated with a median incarceration time of 73 months and 455 (46.4%) were newly sentenced with a median duration of awaiting trial of 15 months. The baseline characteristics of the participants were similar in the two groups (table 1). The majority (914/981[93.2%]) were Black African, the median age was 32 (interquartile range [IQR] 27–37) years and the median number of prisoners per cell was 44 (IQR 41–55). Previous HIV testing was reported by 580/981 (59.1%) and previous tuberculosis treatment by 126/981 (12.8%). Previous tuberculosis treatment was reported more frequently by those currently incarcerated than those newly sentenced (87/526 [16.5%] and 39/455 [8.6%], respectively; p<0.001).

**Table 1:** Characteristics of participants.

Characteristics*		All participants(n = 981)†	Incarcerated ≥6 months(n = 526)‡	Newly sentenced (n = 455)§
**Age (years)**	Median (IQR)	32 (27–37)	34 (30–39)	29 (26–35)
**Country of origin**	South Africa	862 (87.9)	478 (90.9)	384 (84.4)
**Ethnic group**	Black African	914 (93.2)	491 (93.3)	423 (93.0)
**No. of years in school**	Median (IQR)	10 (9–12)	10 (9–12)	10 (8–12)
**Duration of incarceration (months)**	Median IQR)	34 (16–79)	73 (43–102)	15 (6–26)^II^
**Previous incarceration**	Yes	409 (41.7)	232 (44.1)	177 (38.9)
**No. of prisoners per cell** ^(n = 951)^	Median (IQR)	44 (41–55)	44 (41–45)	54 (40–76)
**History of mine work**	Yes	34 (3.5)	18 (3.4)	16 (3.5)
**Health care worker**	Yes	42 (4.3)	19 (3.6)	23 (5.0)
**Previous IVDU**	Yes	14 (1.4)	10 (1.9)	4 (0.9)
**Ever drunk alcohol**	Yes	729 (74.3)	378 (71.9)	351 (77.1)
**Smoking history**	Ex-smoker	190 (19.4)	140 (26.6)	50 (11.0)
	Current smoker	568 (57.9)	254 (48.3)	314 (69.0)
	Non-smoker	223 (22.7)	132 (25.1)	91 (20.0)
**Self-reported previous HIV test**	Yes	580 (59.1)	361 (68.6)	219 (48.1)
**Self-reported previous HIV test result** ^(n = 578)^	Negative	391 (67.6)	239 (66.4)	152 (69.7)
	Positive	168 (29.1)	115 (31.9)	53 (24.3)
	Indeterminate	3 (0.5)	2 (0.6)	1 (0.5)
	Don’t know	16 (2.8)	4 (1.1)	12 (5.5)
**Maxim HIV-1 urine EIA** ^(n = 957)^	Positive	242 (25.3)	132 (25.4)	110 (25.1)
**Self reported current ART use** ^(n = 168)^	Yes	88 (52.4)	60 (52.2)	28 (52.8)
**Previous TB treatment**	Yes	126 (12.8)	87 (16.5)	39 (8.6)

* Data are number of participants (percent) unless otherwise indicated;

† denominator = 981unless otherwise indicated;

‡ denominator = 526 unless otherwise indicated;

§ denominator = 455 unless otherwise indicated;

Duration awaiting trial.

IQR = Interquartile range; IVDU = Intravenous drug use; ART = Antiretroviral therapy; TB = Tuberculosis; No = Number.

### Prevalence of Tuberculosis

At enrolment 10/981 (1.0%) participants were on anti-tuberculous treatment. Three participants did not have enrolment sputum cultures performed and were excluded from further analyses. Of the remaining 968, 374 (38.6%) had at least one clinical feature compatible with tuberculosis, and 337 (34.8%) had a positive WHO symptom screen for people with HIV [25]. Using the study definition, 446/968 (46.1%) were identified as tuberculosis suspects, among whom 332/446 (74.4%) had a one-month follow up visit with repeat investigations.

The case definitions of definite and probable tuberculosis were fulfilled by 28/968 (2.9%) and 6/968 (0.6%) participants respectively, giving a prevalence of undiagnosed tuberculosis of 3.5% ([34/968]; 95% CI 2.4–4.9%). All 6/6 (100%) probable tuberculosis cases were culture positive for *M. tuberculosis.* A further 39/968 (4.0%) fulfilled the case definition of possible tuberculosis, which, if included, would give a prevalence of 7.5% ([73/968]; 95% CI 6.0–9.4%).

The prevalence of undiagnosed tuberculosis (excluding possible cases) was similar in the currently incarcerated and newly sentenced groups (19/491 [3.9%] and 15/438 [3.4%], respectively; p = 0.73). Among participants with undiagnosed tuberculosis, 5/34 (14.7%) were smear positive (any grade). Two participants had 1+ positive smears (one had a single positive smear and the other, two positive smears), two had 2+ positive smears (one had a single positive smear and the other, one 2+ positive smear, and, one 1+ positive smear) and one had a single scanty positive smear. Among isolates from sputum culture, 1/34 (2.9%) had isoniazid monoresistance and 1/34 (2.9%) had resistance to isoniazid and rifampicin.

### Prevalence of HIV

Urine HIV test results were available for 957/981 (97.6%) participants, with 242 testing positive, giving a prevalence of HIV infection of 25.3% (95% CI 22.6–28.2%). The prevalence was similar in the currently incarcerated and newly sentenced groups (132/519 [25.4%] and 110/438 [25.1%], respectively; p = 0.94). The prevalence of HIV infection among those with undiagnosed tuberculosis was 15/34 (44.1%).

### Demographic and Clinical Risk Factors for Undiagnosed Tuberculosis

The risk factor analysis for undiagnosed tuberculosis was restricted to 929/981 (94.7%) participants, excluding 10 on tuberculosis treatment at enrolment, 39 defined as “possible” tuberculosis and three without sputum cultures at enrolment. Urine HIV results were available for 906/929 (97.5%). In the univariable analysis (table 2) undiagnosed tuberculosis was more common in those who had no prior incarceration, shared a cell with >50 prisoners, drank alcohol, were ex-smokers, had HIV infection and reported previous tuberculosis treatment.

**Table 2:** Risk factors for undiagnosed tuberculosis*.

Risk factor/Category	Undiagnosed TBN = 34/929^†‡^		Unadjusted OR (95% CI)	P-value	Adjusted OR^§^(95% CI)	P-value
	Prevalence	%				
**Participant type**						
Incarcerated ≥6 months	19/491	3.9	1.0			
Newly sentenced	15/438	3.4	0.9 (0.4–1.8)	0.72		
**Age (years)**						
20–29	13/351	3.7	1.0	0.98		
30–34	9/237	3.8	1.0 (0.4–2.4)			
>35	12/341	3.5	0.9 (0.4–2.1)			
**Duration of incarceration (years)**						
>3	14/438	3.2	1.0	0.48		
≤3	20/491	4.1	1.3 (0.6–2.6)			
**Previous incarceration**						
No	24/543	4.4	1.0	0.14		
Yes	10/386	2.6	0.6 (0.3–1.2)			
**Number of prisoners per cell ^N = 33/901^**						
≤50	19/631	3.0	1.0	0.12	1.0	
>50	14/270	5.2	1.8 (0.9–3.6)		1.8 (0.9–3.6)	0.12
**History of mine work**						
No	34/896	3.8		0.63^**^		
Yes	0/33	0.0				
**Health care worker**						
No	33/888	3.7	1.0	0.74		
Yes	1/41	2.4	0.6 (0.0–4.1)			
**Intravenous drug use^∫^**						
No	34/918	3.7		>0.99^**^		
Yes	0/11	0.0				
**Ever drunk alcohol**						
No	5/236	2.1	1	0.12		
Yes	29/693	4.2	2.0 (0.8–5.3)			
**Smoking History**						
Current smoker	14/547	2.6	1.0	0.02	1.0	
Ex-smoker	13/170	7.7	3.2 (1.5–6.8)		2.6 (1.2–5.9)	0.07
Non-smoker	7/212	3.3	1.3 (0.5–3.3)		1.3 (0.5–3.2)	
**Maxim HIV-1 Urine EIA^N = 34/906^**						
Negative	19/682	2.8	1.0	0.01	1.0	
Positive	15/224	6.7	2.5 (1.3–5.0)		2.0 (1.0–4.2)	0.06
**Previous TB treatment**						
No	24/826	2.9	1.0	0.003		
Yes	10/103	9.7	3.6 (1.7–7.7)			

* Undiagnosed tuberculosis defined as participants fulfilling the case definition of definite or probable tuberculosis. †Numerator (undiagnosed tuberculosis cases) = 34 unless otherwise indicated. ‡Denominator = 929 unless otherwise indicated (10 participants on tuberculosis treatment at enrolment, 39 “possible” tuberculosis cases and 3 participants without sputum cultures at enrolment were excluded). §Adjusted for number of prisoners per cell, smoking history and HIV status; n = 878 for adjusted model; numerator (undiagnosed tuberculosis) = 33 for adjusted model. **Fisher’s exact test. ∫3 participants reporting previous IVDU use excluded from the analysis (1 without sputum culture at enrolment; 2 with possible tuberculosis).

TB = Tuberculosis; OR = Odds ratio; CI = Confidence interval.

Due to the small number of outcomes (n = 34), the multivariable model was restricted to number of prisoners per cell, smoking history and HIV status, which were most strongly associated with undiagnosed tuberculosis in the univariable analysis. In the adjusted analysis (for n = 878 participants with complete data, table 2) being an ex-smoker (adjusted OR 2.6, 95% CI 1.2–5.9) and HIV infection (adjusted OR 2.0, 95% CI 1.0–4.2) remained associated with undiagnosed tuberculosis.

### Sensitivity, Specificity and Predictive Values of Screening Tools for Undiagnosed Tuberculosis

Using definite and probable tuberculosis as our gold standard, the performance of different screening modalities was evaluated for 867/981 (88.4%) participants who had complete data for all symptoms and investigations (table 3).

**Table 3:** Performance of screening tools for undiagnosed tuberculosis* (N = 867).

Screening tools:	Prevalence n(%)(N = 867)^†^	Sensitivity(N = 34)	Specificity(N = 833)	PPV	NPV
Cough >2 weeks	76/867 (8.8)	23.5	91.8	10.5	96.7
Any cough	182/867 (21.0)	35.3	79.6	6.6	96.8
Night sweats	112/867 (12.9)	5.9	86.8	1.8	95.8
Fever	89/867 (10.3)	14.7	89.9	5.6	96.3
Self-reported weight loss	132/867 (15.2)	17.7	84.9	4.6	96.2
Symptom combination^‡^	227/867 (26.2)	29.4	74.0	4.4	96.2
WHO tool^§^	298/867 (34.4)	38.2	65.8	4.4	96.3
CXR features suggestive of TB	89/867 (10.3)	70.6	92.2	27.0	98.7
Any CXR abnormality^∫^	146/867 (16.8)	73.5	85.5	17.1	98.8
**Combination of screening tools:**					
Cough >2weeks or CXR features suggestive of TB	151/867 (17.4)	73.5	84.9	16.6	98.7
Any cough or CXR features suggestive of TB	245/867 (28.3)	79.4	73.8	11.0	98.9
Symptom combination^‡^ or CXR features suggestive of TB	286/867 (33.0)	73.5	68.7	8.7	98.4
WHO tool^§^ or CXR features suggestive of TB	349/867 (40.2)	79.4	61.3	7.7	98.7

Data are percentages unless otherwise indicated. *Undiagnosed tuberculosis defined as participants fulfilling the case definition of definite or probable tuberculosis. † Denominator = 867 unless otherwise indicated (10 participants on tuberculosis treatment at enrolment, 39 “possible” tuberculosis cases, 3 participants without sputum cultures at enrolment and 62 with missing chest radiographs at enrolment were excluded). ^∫^Any CXR abnormality = active tuberculosis 89 (61.0%); previous tuberculosis 23 (15.8%); trauma/bullets 16 (11.0%); abnormal cardiac silhouette 9 (6.2%); other 9 (6.2%). ^‡^Any of cough >2 weeks, night sweats or unintentional weight loss. ^§^Any of current cough, fever, unintentional weight loss or night sweats (WHO symptom screen for people with HIV [25]). NPV = negative predictive value; PPV = positive predictive value; WHO = World Health Organization; CXR = Chest radiograph; TB = tuberculosis.

The sensitivity of individual symptoms (cough of any duration; cough >2 weeks; night sweats; fever or unintentional weight loss) was low, ranging from 5.9% for night sweats to 35.3% for cough of any duration (table 3). Specificity ranged from 79.6% for cough of any duration to 91.8% for cough >2weeks. Sputum smear had poor sensitivity (14.7%). The sensitivity and specificity of chest radiographic features suggestive of tuberculosis was 70.6% and 92.2% respectively. While any chest radiographic abnormality had a higher sensitivity (73.5%), specificity was lower (85.5%). A high proportion of abnormal chest radiographs (11.0%) in this population had evidence of trauma/bullets.

Combining symptoms (either any of cough >2weeks, night sweats or unintentional weight loss [henceforth known as the symptom combination]; or the WHO symptom screen for people with HIV [25]) increased the sensitivity (29.4% and 38.2% respectively), but at the cost of specificity (74.0% and 65.8% respectively). A screening tool combining one or more symptoms with chest radiographic features suggestive of tuberculosis increased the sensitivity to >73% (table 3). The most sensitive combinations (with a sensitivity of 79.4%), were adding chest radiographic features suggestive of tuberculosis to cough of any duration or the WHO symptom screen; however, again, specificity was reduced (73.8% versus 61.3% respectively). All combinations of symptoms and sputum smear (data not shown) had poor sensitivity (<42%).

The performance of screening tools was assessed according to HIV status for 846/867 (97.6%) participants who had HIV test results (see Table 4). When stratified by HIV status, the combination of cough of any duration and/or chest radiographic features suggestive of tuberculosis still gave the highest sensitivity and specificity in both HIV-negative and HIV-positive participants (sensitivity 84.2% versus 73.3% respectively and specificity 75.4% versus 70.2% respectively).

**Table 4:** Performance of screening tools for undiagnosed tuberculosis* stratified by HIV status (N = 846).

Screening tools:	Prevalence (N = 846)^†^		Sensitivity (N = 34)		Specificity (N = 812)		PPV		NPV	
	HIV-N n = 633	HIV-P n = 213	HIV-N n = 19	HIV-P n = 15	HIV-N n = 614	HIV-P n = 198	HIV-N	HIV-P	HIV-N	HIV-P
Cough >2 weeks	7.6	12.7	21.0	26.7	92.8	88.4	8.3	14.8	97.4	94.1
Any cough	19.4	24.9	31.6	40.0	80.9	76.3	4.9	11.3	97.4	94.4
Night sweats	10.9	18.3	0.0	13.3	88.8	81.3	0.0	5.1	96.6	92.5
Fever	8.9	13.6	10.5	20.0	91.2	86.9	3.6	10.3	97.0	93.5
Reported weight loss	13.4	20.7	10.5	26.7	86.5	79.8	2.3	9.1	96.9	93.5
Symptom combination^‡^	23.5	34.3	21.0	40.0	76.4	66.2	2.7	8.2	96.9	93.6
WHO tool^§^	31.1	43.7	31.6	46.7	68.9	56.6	3.0	7.5	97.0	93.3
CXR suggestive of TB	9.3	13.1	79.0	60.0	92.8	90.4	25.4	32.1	99.3	96.8
Any CXR abnormality	15.3	20.2	79.0	66.7	86.6	83.3	15.5	23.3	99.3	97.1
**Combination of screening tools:**										
Cough >2 weeks or CXR suggestive of TB	15.5	23.5	79.0	66.7	86.5	79.8	15.3	20.0	99.2	96.9
Any cough or CXR suggestive of TB	26.4	32.9	84.2	73.3	75.4	70.2	9.6	15.7	99.4	97.2
Symptom combination^‡^ orCXR suggestive of TB	30.2	41.3	79.0	66.7	71.3	60.6	7.9	11.4	99.1	96.0
WHO tool^§^ or CXR suggestive of TB	37.1	48.8	84.2	73.3	64.3	53.0	6.8	10.6	99.2	96.3

Data are percentages. * Undiagnosed tuberculosis defined as participants fulfilling the case definition of definite or probable tuberculosis. † Denominator = 846 unless otherwise indicated (10 participants on tuberculosis treatment at enrolment, 39 “possible” tuberculosis cases, 3 participants without sputum cultures at enrolment, 62 with missing chest radiographs at enrolment and 21 with missing urine HIV test results were excluded). ^‡^Any of cough ≥2weeks, night sweats or unintentional weight loss. ^§^Any of current cough, fever, weight loss or night sweats (WHO symptom screen for people with HIV [25]). NPV = negative predictive value; PPV = positive predictive value; HIV-N = HIV-negative; HIV-P = HIV-positive; WHO = World Health Organization; CXR = Chest radiograph; TB = tuberculosis.

## Discussion

As far as the authors are aware, this study is the first to systematically evaluate, using a symptom screen, chest radiography and sputum microscopy and culture, the prevalence of active pulmonary tuberculosis, and, HIV in a representative sample of male prisoners in Sub-Saharan Africa. The prevalence of undiagnosed culture-positive tuberculosis was high at 3.5%, but would rise to 7.5% if those with typical chest radiographs or scanty sputum smears, but negative cultures, were included.

Direct comparison with other prison prevalence surveys from Sub-Saharan Africa, which report tuberculosis prevalences ranging from 1 to 6% [2], [4], [6]–[12] (3% at a prison in KwaZulu-Natal South Africa [16]), is difficult, because of differing sampling strategies, screening methodologies and case definitions. The prevalence of tuberculosis found in our study is higher than the estimated prevalence for the general population (0.8% in 2010 [14]), and, the mines (approximately 2.2% in 2010 [30]), in South Africa. Results consistently show a higher prevalence of undiagnosed tuberculosis in prisons compared to the general population, calling for urgent measures to address this. Untreated tuberculosis among prisoners is not only a risk to other prisoners; it also has wider implications for the general population through transmission to prison staff, visitors, and communities after release [1].

Only 14.7% of prisoners with undiagnosed tuberculosis were smear positive. Forty five percent of all notified pulmonary tuberculosis cases in South Africa in 2010 were smear positive (WHO 2011). This is in keeping with studies that show a lower proportion of cases identified through active case finding are smear positive, when compared with those identified through passive case finding [31]–[33].

Identifying prisoners with active tuberculosis through screening, and, commencing appropriate treatment, will make infectious patients non-infectious. This should be coupled with encouraging self-referral and infection control strategies such as isolating infectious tuberculosis cases [1], [2], [21].

If high risk groups were identifiable, screening for tuberculosis could be targeted. Factors independently associated with undiagnosed tuberculosis were being an ex-smoker and HIV infection. Previous tuberculosis was strongly associated with undiagnosed tuberculosis in the univariable analysis. The association with previous tuberculosis has been reported in prison studies from Cameroon [4], Bangladesh [34] and Brazil [26]. However, only 29% of prisoners with undiagnosed tuberculosis had previous tuberculosis in our study. Studies from prison settings have not previously demonstrated an association between tuberculosis and being an ex-smoker. However, given that this is a cross-sectional study, reverse causality cannot be excluded. While the association between tuberculosis and HIV infection is well known, it has not been widely reported on in prison studies mainly due the lack of systematic screening for HIV infection. In a study among prisoners in Cameroon [4], HIV infection was associated with prevalent tuberculosis (including those on treatment) but not with undiagnosed prevalent tuberculosis. HIV infection is less strongly associated with prevalent compared with incident tuberculosis [35] and may be a less important risk factor in prisons, where the risk among HIV negative people is also high, compared with the general population. Our results to a certain extent corroborate this finding; the odds of HIV infection in those with undiagnosed tuberculosis was relatively low. In addition, screening based on HIV status would miss a large proportion of undiagnosed tuberculosis patients; the prevalence of HIV infection in those with undiagnosed tuberculosis was 44.1% and in practice HIV status of all prisoners would not be known. Therefore in this setting, the risk of tuberculosis is not limited to conventional at risk groups, necessitating universal screening.

The proportion of participants with undiagnosed tuberculosis was similar among newly sentenced and currently incarcerated prisoners. Newly sentenced prisoners had spent a median duration of 15 months awaiting trial prior to being sentenced. Therefore the two groups studied were more homogenous in terms of duration of incarceration than originally anticipated at the start of the study. In most penal systems, people may remain in detention facilities for long periods. In addition, on univariable analysis duration of incarceration was not associated with undiagnosed tuberculosis. Therefore, we recommend screening for all those entering the prison system, and periodic rescreening of the incarcerated population. Newly sentenced prisoners with an expected stay of less than three months were excluded from the study. However, the median duration that newly sentenced prisoners had awaited trial reflects prolonged exposure to the penal system prior to being sentenced. This justifies recommendations for screening all those entering the prison system.

Our data suggests that chest radiography in combination with cough of any duration should be used to screen new entrants to prisons and the incarcerated population. Likewise, chest radiography in combination with cough of any duration should be used to screen both HIV positive and HIV negative prisoners. Due to the small number of smear positive cases, we were unable to investigate the role of screening tools in identifying smear positive cases.

Our results support CDC [36] and WHO [21] guidance, which recommends chest radiography in addition to standardised symptom screening for all prisoners entering high tuberculosis risk prisons. Data from Brazil [24], [26] and Hong Kong [27] also support the utility of chest radiography for screening in prisons. The positive predictive value of the combination of chest radiographic features suggestive of tuberculosis and cough of any duration in our study was 11.0%; for approximately every 10 people investigated further, one will be found to have tuberculosis. This is in line with recommendations for sputum microscopy [37]. Mass miniature radiography for new entrants has been implemented in US detention centres at a cost of only $3 per prisoner screened and found to increase case finding and speed up isolation of tuberculosis cases [38], [39]. However, the logistics of conducting chest radiographic screening within prisons, including the staffing and expertise required, maintenance of equipment and movement of prisoners, also needs to be considered if implementation is planned. Modelling studies in prisons support the effectiveness of annual chest radiographic screening (with and without symptom screening) in decreasing tuberculosis prevalence, and, its cost effectiveness [40], [41]. However, model settings and assumptions may limit the generalisability of findings to high tuberculosis and HIV burden settings, necessitating further work in this area.

The role of new diagnostic tests for tuberculosis, in particular the role of the Xpert MTB/RIF, requires full assessment in this population. With reported overall sensitivities ranging from 73.3% to 97.6%, the Xpert MTB/RIF has been evaluated as both a diagnostic and screening tool [42], [43]. The rapid identification of patients with drug resistant tuberculosis, with timely commencement of treatment, would also help to prevent the spread of drug resistant disease [43]. Following WHO endorsement, it is being widely rolled out in South Africa, aiming to replace smear microscopy [42], [43]. Identifying whom to test with the Xpert MTB/RIF, given the low sensitivity of symptoms, is still a challenge. The cost per Xpert MTB/RIF test is greater than smear microscopy. However, given the high specificity even in the context of a prevalence survey [44], it could potentially be used as a screening and diagnostic test simultaneously, which could have overall cost-savings by limiting the number of investigations needed per diagnosis, particularly given the recent decrease in price of the Xpert MTB/RIF test. A screening study conducted among HIV positive prisoners in Malaysia using a single sample of unprocessed sputum found a sensitivity of 53% and a specificity of 100% [45]. Further work is required to investigate test characteristics among all prison populations, including using different screening algorithms. Modelling studies from prisons in the former Soviet Union, community ART programmes in South Africa and diagnostic services in India and Sub-Saharan Africa suggest that screening and diagnosis of tuberculosis with the Xpert MTB/RIF is cost-effective [40], [46], [47], although the limited generalisability of findings to prisons in Sub-Saharan Africa warrants further work in this area.

Anonymised HIV testing was undertaken to ensure high uptake to testing, to enable tuberculosis results to be fully interpreted. The prevalence of HIV infection among prisoners was high at 25%, arguing for routine offer of HIV testing with linkage to care, as well as active case finding for tuberculosis, tuberculosis infection control measures and scale-up of isoniazid preventive therapy usage [22], [48]. However, the lower specificity of the HIV urine EIA could have resulted in an overestimation of HIV prevalence, which should be considered when interpreting results.

Strengths of our study include representative sampling and high uptake into the study. Limitations include incomplete follow up of tuberculosis suspects, which could have resulted in missed tuberculosis diagnoses: thus the prevalence of undiagnosed tuberculosis of 3.5% is a minimum estimate; indeed if cases classified as “possible” (based on chest radiographic features highly consistent with tuberculosis and scanty sputum smears) had been included, the prevalence of undiagnosed tuberculosis could be as high as 7.5%.

## Conclusions

We found a high prevalence of undiagnosed tuberculosis in this South African prison, justifying universal screening for new entrants to the facility and periodic rescreening of those incarcerated. Our data suggest a screening tool comprising cough of any duration and chest radiography; further work is needed to establish how best to use new tests for tuberculosis in screening and diagnostic algorithms in this setting.
